# Novel read density distribution score shows possible aligner artefacts, when mapping a single chromosome

**DOI:** 10.1186/s12864-018-4475-6

**Published:** 2018-02-09

**Authors:** Fedor M. Naumenko, Irina I. Abnizova, Nathan Beka, Mikhail A. Genaev, Yuriy L. Orlov

**Affiliations:** 1 Novosibirsk State University, Pirogova, 1, Novosibirsk, 630090 Russia; 2 Wellcome Trust Sanger Institute, Cambridge, UK; 3 Babraham Institute, Cambridge, UK; 4 University of Hertfordshire, Hertfordshire, UK; 5 Institute of Cytology and Genetics SB RAS, Novosibirsk, Russia; 6 Institute of Marine Biology Researches of RAS, Sevastopol, Russia

**Keywords:** Next-generation sequencing, DNA alignment, Read density distribution

## Abstract

**Background:**

The use of artificial data to evaluate the performance of aligners and peak callers not only improves its accuracy and reliability, but also makes it possible to reduce the computational time. One of the natural ways to achieve such time reduction is by mapping a single chromosome.

**Results:**

We investigated whether a single chromosome mapping causes any artefacts in the alignments’ performances. In this paper, we compared the accuracy of the performance of seven aligners on well-controlled simulated benchmark data which was sampled from a single chromosome and also from a whole genome.

We found that commonly used statistical methods are insufficient to evaluate an aligner performance, and applied a novel measure of a read density distribution similarity, which allowed to reveal artefacts in aligners’ performances.

We also calculated some interesting mismatch statistics, and constructed mismatch frequency distributions along the read.

**Conclusions:**

The generation of artificial data by mapping of reads generated from a single chromosome to a reference chromosome is justified from the point of view of reducing the benchmarking time. The proposed quality assessment method allows to identify the inherent shortcoming of aligners that are not detected by conventional statistical methods, and can affect the quality of alignment of real data.

## Background

In the last few years next-generation sequencing (NGS), also known as high-throughput sequencing, has shown an impressive amount of development due to significantly increased throughput accompanied by plunging costs. The production of gigabytes of sequencing data in a few hours presents ever-increasing demands on the quality and speed of processing [1].

Mapping reads against a reference genome is typically the first step in analyzing next-generation sequencing data. It is an important step for further analysis, such as identification of protein binding sites from ChIP-sequencing data or variant calling. The quality and efficiency of mapping becomes critical. Hence the growing interest in benchmarking of short read aligners is clear.

A number of robust benchmarking surveys of short read aligners have been published [2–9]. These surveys focus on evaluating of such aspects of mapping accuracy as number of correctly and incorrectly mapped reads, percentage of multi-mapped hits (it will be explained later in the text), variation and errors (single nucleotide polymorphism, splicing, inserts and deletions rate etc.), and technical features (execution time and random-access memory). To reliably estimate these tools, simulated data is often used because of their predictability, reproducibility and manageability.

In particular, manageability is manifested in the fact that artificial sequence can be constructed on the basis of a single chromosome. It is natural to assume that the aligning of such data requires time that is reduced as minimum in proportion to the ratio of the chromosome length to the whole genome length. Moreover, further processing (peak calling, etc.) is performed independently for each chromosome. As a consequence, the results of one chromosome treatment reflect the accuracy and efficiency of the software algorithm in a relevant way, with a proportional total reduction in time.

In this article, we assessed the difference between alignment of reads generated from a single chromosome against a corresponding reference chromosome, and against an entire reference genome. Then we generated reads from a whole genome, and compared their distribution with the above cases. We also compared the proportion of mismatched reads in each case above, and visualized the mismatches’ distributions.

All the cited studies were carried out using statistical metrics based on the count of correctly, incorrectly and unmapped reads. As we discovered, such metrics are not sufficient for a comprehensive assessment of the mapping quality. They do not take into account how well the recovered alignment repeats the initial distribution of the read density. According to our measurements, wrongly mapped full-defined reads range from 0.2% to 3.8%, and lost reads range from 0% to 9.5% of the total number, depending on the software and conditions. In terms of repetition of the distribution density, such a slight fraction can be neglected, but only if these reads are distributed evenly. If ‘wrong‘reads for some reason are assembled into regions with heightened read density, or lost and matched to other chromosomes reads were initially located in compact regions of the original sequence, it may cause biases and corresponding wrong conclusions. To take into account the influence of this factor, we introduced a new metric, and showed its practical significance.

## Methods

### Synthetic data

For our purposes, we needed a simulator of uniformly distributed DNA reads with the following requirements: (i) all reads should be fully defined, (ii) it should generate the sequence in two formats: in FastQ as an initial data for the verified aligner, and in SAM/BAM/BED as a reliable comparison template (‘precise alignment’). Under a fully defined read, we mean a read that (a) carries information in its original actual position, (b) has the maximum quality value, (c) does not include any ambiguous reference characters (‘N’). Such set of reads may be called ‘ideal’.

There is a wide choice of simulators for genomic next generation sequencing. A brilliant and almost exhaustive review of more than twenty of them was carried out by M.Escalona et al. [10]. We also found two simulation-related sources, not included in the review: **MAQ** [11] and **SEAL** [12].

However, none of the considered tools meets our requirements to the fullest. Therefore we chose the ChIP-seq simulator **isChIP** [13], which is able (in the ‘control’ mode) to generate the sequences that completely meet our needs. The program was used with default settings, the only exception was the refinement of the ‘number of cells’ option, which was 10 (for single end mode) and 5 (for paired end one).

Our dataset was derived from control test (‘input’) generated by **isChIP** on UCSC mouse reference genome (mm9), with the length of reads stated as 50 bp.

To approximate real life condition, the first data set is composed of almost 4 million reads randomly and uniformly drawn from the reference chromosome 1.

The second data set consist of 51 million reads drawn from the whole reference genome.

As we have the ‘precise alignment’ with known locations of every drawn read by simulation, let us call this sample the ‘Gold Standard Alignment’ (GSA), ‘taking a leaf out of book’ [9].

### Implementation

We applied our benchmarking tests on 7 open source DNA sequencing mapping tools, namely **Bowtie** (1.1.1) [14], **Bowtie2** (2.2.4) [15], **BWA** (0.7.5 and 0.7.12 applying two algorithms) [16], **MAQ** (0.7.1) [11], **MOSAIK** (2.2.3) [17], **SMALT** (0.7.6) [18]. Another 2 aligners have been rejected after repeated trials: **BBMap** (36.32) and **gnumap** (3.0.2). We also tried to compile **BLASR**, **GSNAP** and **STAR**, but without success.

The default settings were used for the software mentioned above. The only exception was an increase in memory per thread up to 800 mb in case of **Bowtie**.

Commonly, methods based on binary classification: ‘aligned’ vs ‘non-aligned’ reads, are used to assess mapping accuracy. Indeed as we have a GSA we are able to quantify the aligned reads by computing so called ‘confusion matrix’ of performance: amount of true positives (TP), reads aligned correctly; false positives (FP), reads aligned wrongly; false negatives (FN), reads not aligned but belonging to the simulated GSA. The true negatives (TN) are legitimately rejected reads, they are a degenerate case in this context. We can then calculate different statistical measures, derived from confusion matrix (such as mapping accuracy and recall), to evaluate the performance of mappers.

However, not only these statistical measures are important in assessing of an aligner’s performance, but also an aligned reads’ density distribution, which reflects evenness and variance of a coverage. We want to check whether the recovered (obtained after alignment) read density is similar to the original one.

Therefore, for the most comprehensive evaluation of the mapping accuracy, two complementary measures should be used: the measure of robustness/accuracy is one, and the measure of distribution similarity as another.

### Measure of test accuracy

As a measure of test accuracy we will use the F_1_ value, following the example of Farzana et al. [9].

The F_1_ value can be interpreted as a weighted average of the precision and recall:

$$ {\mathrm{F}}_1=2\times \left(\frac{p\times r}{p+r}\right) $$

where *p* denotes the positive predictive value (precision), and r denotes the true positive rate (sensitivity, or recall). The F_1_ value varies between 0 and 1, indicating highest accuracy when it is 1. Precision and recall are computed from the confusion matrix according to: *p* = TP/(TP + FP), *r* = TP/(TP + FN).

Note that we interpreted TP, FP and FN definitions in a slightly different way than Farzana et al. While the authors defined them in terms of true or wrong location only, we also took into consideration the mismatches per read, i.e. the number of different nucleotides as compared with referenced fragment.

Specifically, we defined *true positives* as a number of all reads without mismatches, not only reads mapped exactly to the same position as prescribed by GSA. The hits without mismatches, but mapped on wrong positions are the cases of multiple mappings due to paralogous sequences in the reference genome, so-called multi-map reads, or simply multi-reads.

Although most of the tested tools reveal multi-reads and assign them a low-quality value, it is impossible to identify which short sequence between two identical is ‘true ‘. As a result, some of the exactly matched reads also belong to multi-map and are awarded by low value. In our measurements the number of such detected ‘wrong-placed‘can reach 3.5% of the total initial number, and about the same percentage of low-quality reads had been included in the ‘right-placed‘group. So, to achieve the fair evaluation, all multi-reads should be either included or excluded from the TP enumeration. Here we include multi-reads.

Accordingly, we assigned *false positive* as the number of reads with mismatches and mapped to a different position than the one defined by the global alignment. We will call them simply mismatched reads.

*False negative* is the number of lost reads, i.e. reads rejected by the aligner due to using heuristics in the mapping algorithm or limitations of the default options.

### Measure of distribution similarity

As a measure of the distributions’ similarity we will use a *relative standard deviation of read density distribution from sample*, CVS.

We defined the ‘read density distribution’ (or simply ‘density profile’) as a discrete set of numbers (frequencies) of the base adjacent regions on the reference chromosome (windows), containing 1, 2, 3, etc. reads. To avoid windows with meaningless density, before splitting the sequence into windows, we removed all the gaps (i.e. regions in a reference chromosome completely filled with ambiguous reference characters ‘N’) from it. In our experiments, the length of the window was 200 bp, the minimum length of the gap was 50 bp. Besides, the read was considered belonging to window if it’s centre was placed within the window. Such adjustment allows to keep the total amount of reads permanent, so we obtain the actual density.

The density profiles were computed for the original sequence and for the results of each aligner.

To compare these profiles we define an analogue of coefficient of variation, using GSA’s counts in a window, assuming it is what we expect from a good aligner’s job:

$$ \mathrm{CVS}=\frac{1}{\upmu}\sqrt{\frac{1}{\mathrm{N}}{\sum}_{\mathrm{i}=1}^{\mathrm{N}}{\left({\mathrm{x}}_{\mathrm{i}}-{\mathrm{X}}_{\mathrm{i}}\right)}^2}, $$

Here CVS denotes the ‘coefficient of variation of a sample‘, x_i_ is the number of windows containing i reads obtained by an aligner, X_i_ denotes the number of GSA windows containing i reads, μ denotes the GSA’s value across all window frequencies, N is the total number of window frequencies. The minimum value CVS = 0 means no deviation from the GSA. The maximum value is not limited.

All the computational work was carried out by our software **DenPro** [19].

As we mentioned before, first of all we are interested in ‘false gaps‘and ‘false peaks‘. The increased frequency of windows with a small number of reads means the abundance of deeps, while the increased frequency of windows with a large number of reads indicates the falsely enriched areas.

‘False gaps‘ and ‘false peaks‘ appear as a deviation of the density profiles after aligning from the initial in the range of window counts with a small number of reads and with large ones, respectively. In our data, zero-numbered windows were missing, although with smaller densities characteristic of ‘poor‘ experimental data and a shorter window length, they may well be present. The observed deviations of the restored density profiles for the windows with a small number of reads, were limited to the frequency of windows containing a single read. We called this singular range ‘head’. Deviations for the windows with a large number of reads have become noticeable when frequency was 13 reads per window or more. We called this range ‘tail’. For a numerical evaluation of these phenomena, it makes sense to calculate the coefficient of variation in these ranges separately, along with the total CV. Respectively, we called these coefficients ‘head CVS’, CVSh, and ‘tail CVS’, CVSt:

$$ \mathrm{CVSh}=\frac{1}{\mu}\sqrt{{\left({x}_1-{X}_1\right)}^2},\kern0.5em \mathrm{CVSt}=\frac{1}{\mu}\sqrt{\frac{1}{N-12}{\sum \limits}_{i=13}^N{\left({x}_i-{X}_i\right)}^2} $$

Here μ denotes the GSA’s value across the specified ranges.

Three series of tests have been conducted: (i) mapping of reads from a single chromosome to a reference chromosome, (ii) mapping of reads from a single chromosome to a reference genome, and (iii) mapping of reads from a whole genome to a reference genome. For convenience, let us call these series *case1*, *case2* and *case3*.

*Case1* is refined and unpractical, but it encourages software to produce the highest possible, ‘ideal‘result, and could be very appealing for the benchmarking of aligners and peak callers.

*Case2* is unpractical too, it is interesting as a light candidate for the benchmarking of peak callers.

*Case3* corresponds to real life conditions.

## Results

### Test distribution similarity with GSA.

#### *Case1*: Mapping of reads from single chromosome to reference chromosome

**Bowtie** and **Bowtie2** have been excluded from this case since they are supplemented by pre-build reference genome indexes. We also evaluated **BWA** 0.7.5 in this series only, mainly to demonstrate the difference between the old and latest versions of program. Hereinafter we used the latest version **BWA** 0.7.12 as more accurate. Wherever we mention **BWA** without version indication we refer to **BWA** 0.7.12.

**Fig. 1:**
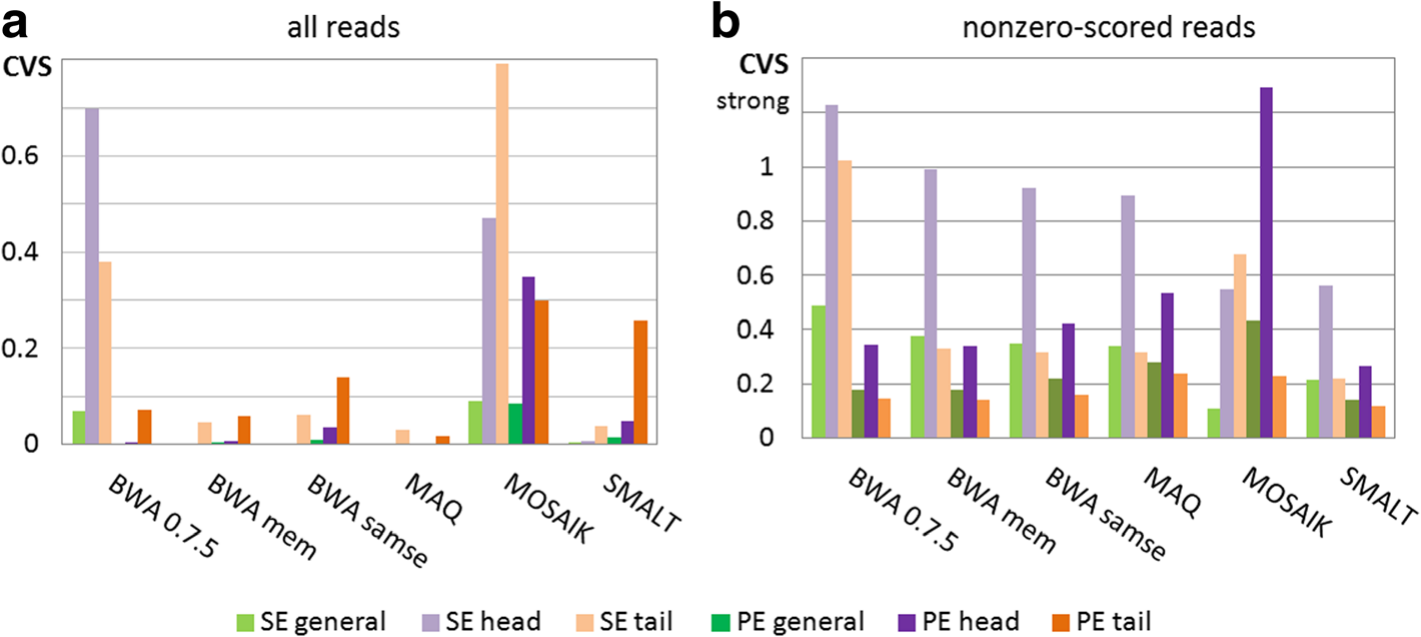
Coefficients of variation of the sample, *case1*. Panel labels: general (total) CVS are marked in green, head CVS - in mauve, tail CVS - in brown. SE are shown with light colour bars, PE – with dark colour bars

CVSs for *case1* are shown in Fig. 1A. We can observe the very different level of CVS for different aligners. Only **MAQ**, and partly **BWA-mem** showed the perfect coincidence with the original read density distribution in both single end (SE) and paired end (PE) modes. In SE mode **SMALT** and partly **BWA-samse** also showed the good results, while **BWA 0.7.5** and **MOSAIK** demonstrated a high level of unexpected depleted and enriched regions, accordingly. In PE mode however, the picture is changed: **MOSAIK** showed a heightened level of ‘false peaks‘and ‘false gaps’**,** and **SMALT** – a heightened level of ‘false peaks‘, unlike other tools.

**Fig. 2:**
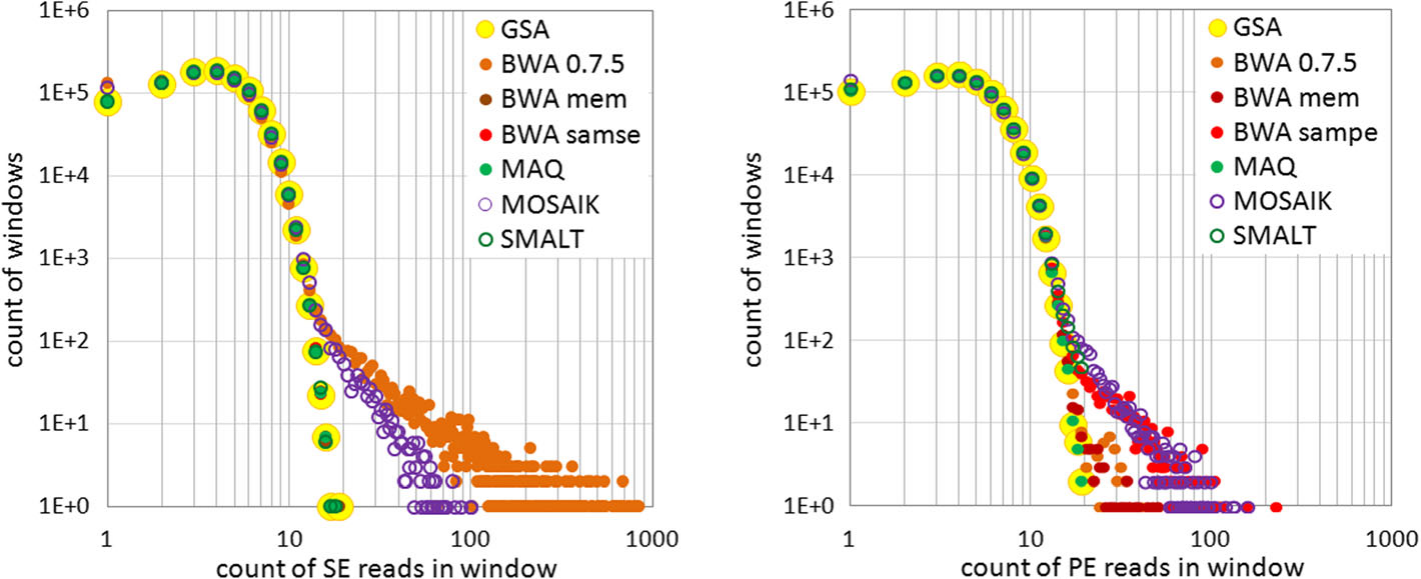
Density profiles for mapping of reads in log-log coordinates, *case1*

**Fig. 3:**
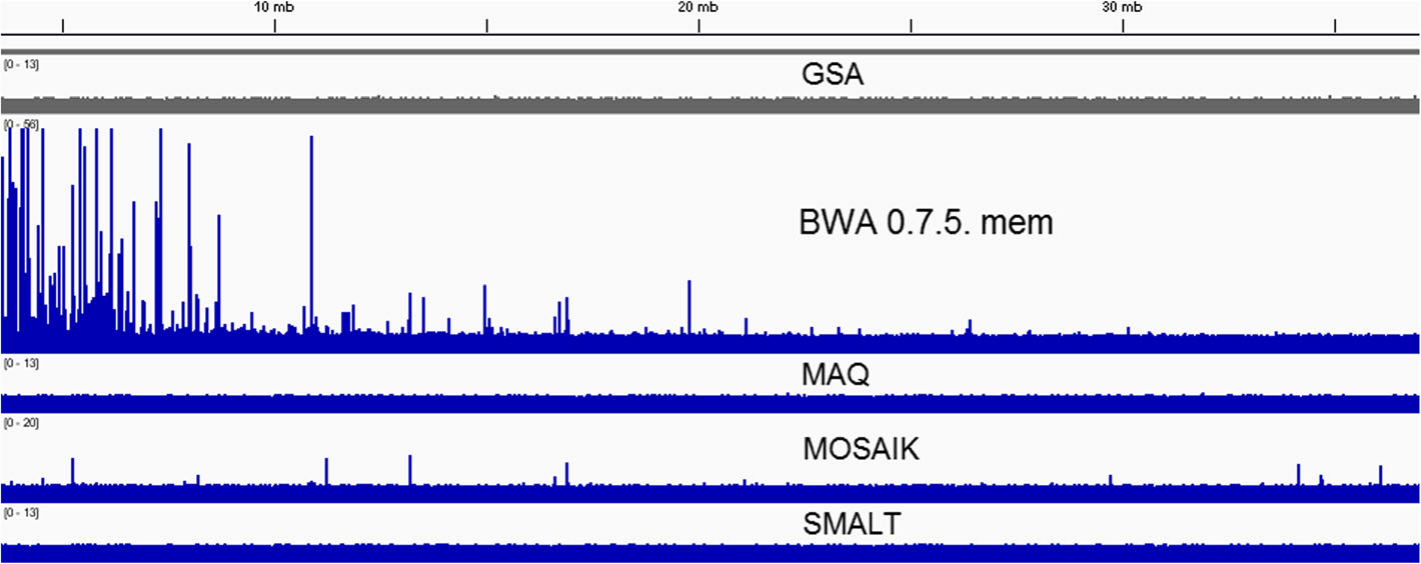
Fragment of coverage of SE alignments, *case1*. All the tracks have the same data range (vertical scale)

As an illustration of these issues, we give direct combined images of SE and PE density profiles (Fig. 2). Falsely enriched regions of the two aligners become obvious in the fragment of coverage graph (Fig. 3). In case of **BWA 0.7.5** these ‘false peaks‘are concentrated at the beginning of the chromosome, while **MOSAIK** produces such enrichments along the entire chromosome.

But these results should be considered as insufficient without taking into account the alignment quality score.

We found that more than 90% of exactly matched SE reads had the highest possible score for all examined aligners (except **MOSAIK** with its value of 66%). For PE reads the same minimum value was 94% (15% in case of **MOSAIK)**. Only **Bowtie** marks all its hits by the maximum score value. **Bowtie2** and **MAQ** assign the lowest possible quality score to all, **BWA** – to almost all of mismatched and multi- hits. **MOSAIK** and **SMALT** mark mismatched and multi- reads with low scores.

All the aligners, except **Bowtie2**, assign zero score for the most doubtful located reads. It is interesting to know what effect such zero-scored hits have on the density profile. We called ‘reliable’ sequences with filtered zero-scored reads, and represented them in Fig. 1B.

#### *Case2*: Mapping of reads from single chromosome to reference genome

**Fig. 4:**
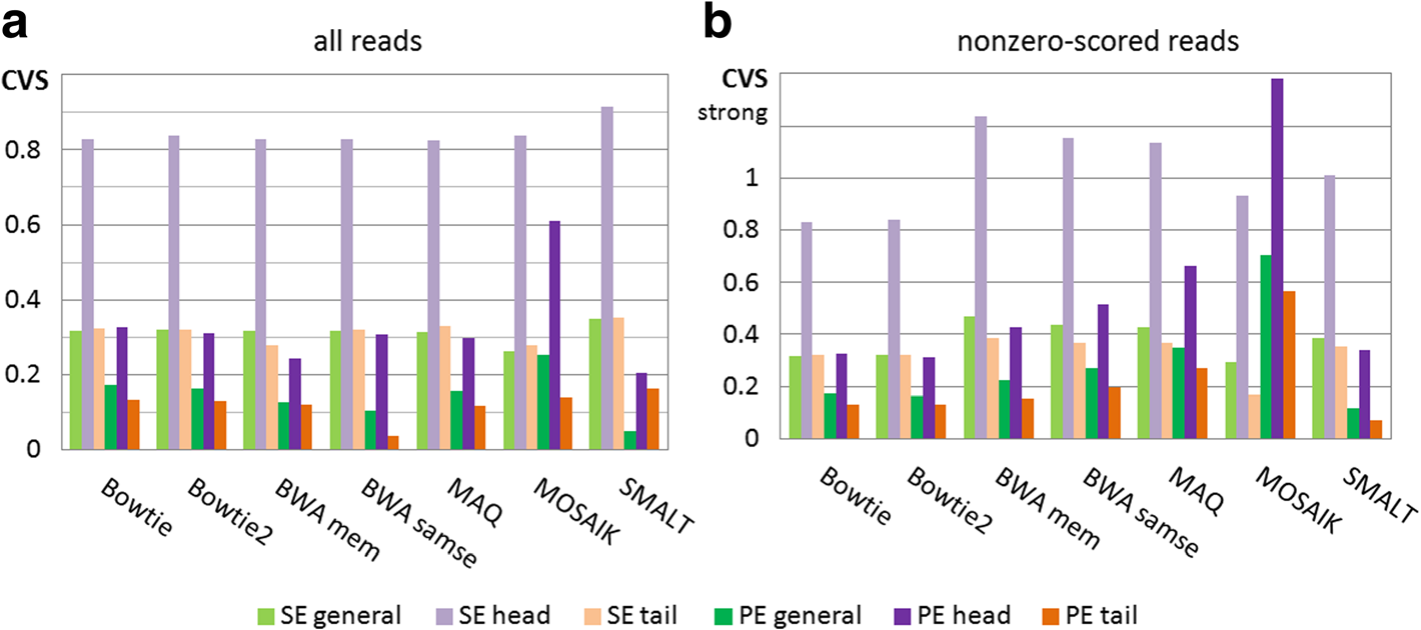
Coefficients of variation of the sample, *case2*. Panel labels: general (total) CVS are marked in green, head CVS - in mauve, tail CVS - in brown. SE are shown with light colour bars, PE – with dark colour bars

**Fig. 5:**
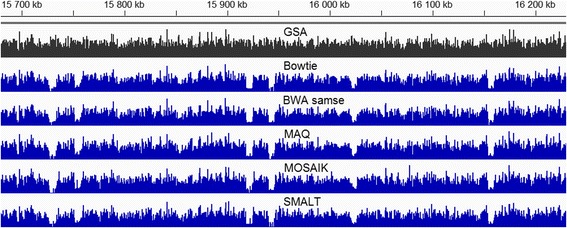
Fragment of coverage of PE alignments, *case2*

There is a dramatic change in coverage evenness when aligners map the same data (simulated from a single chromosome) to the whole genome (see Fig. 4A). We can observe an almost equal pattern of synchronous read-depleted regions (gaps) for all aligners. The gaps have characteristic length of 1–10 kpb (see Fig. 5). And the difference between ordinary and ‘reliable’ distributions became much less (Fig. 4B).

According to CVS measures, in this case all aligned sequences are distinguished by a remarkable similarity between themselves, in contrast to other cases. This is also confirmed by the Pearson correlation: the cross-correlation coefficients are about 0.98, while in the other cases they vary from 0.89 to 0.93 (with the exception of **MOSAIK**, which shows the lower value for all cases). Pearson coefficients were calculated by our software **bioCC** [20] and are presented in supplementary tables, along with the remaining results.

#### *Case3*: Mapping of reads from genome to reference genome.

As we mentioned above, this is the most realistic condition, and consequently the most complicated case.**MAQ** and **MOSAIK** have been excluded from this series due to the requirement of inappropriate execution time (more than 50 CPU hours on a high- performance computer). Besides **MAQ** demands additional efforts to split the job because of limited input sequence size (2–3 million reads).

**Fig. 6:**
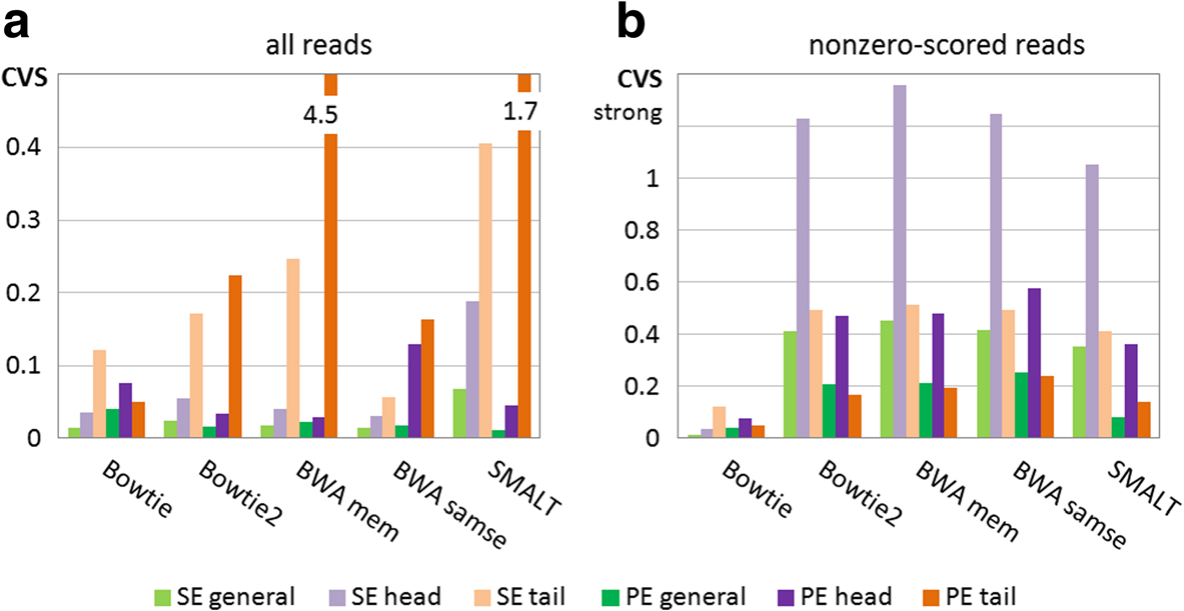
Coefficients of variation of the sample, *case3*. Panel labels: general (total) CVS are marked in green, head CVS - in mauve, tail CVS - in brown. SE are shown with light colour bars, PE – with dark colour bars

As shown in Fig. 6A, only **Bowtie** demonstrates a very good coincidence with GSA in both SE and PE modes. **BWA** and **SMALT** showed an unexpected high level of false PE ‘peaks‘. This time, **SMALT** showed a relative clustering of ‘false peaks‘ at the first third of the chromosome, while **BWA** generates such peaks evenly along the chromosome. A low ‘reliable’ CVS levels for **Bowtie** should not be misleading, since this aligner does not differentiate the quality score (Fig. 6B).

Note that all calculations were performed for the first chromosome. However, to avoid possible dependency on the chromosome particular structure, we conducted control measurements for two more chromosomes (second and third) in a limited conditions (*case1* and *case2* in a single end mode), and obtained very consistent results.

### Test accuracy

**Fig. 7:**
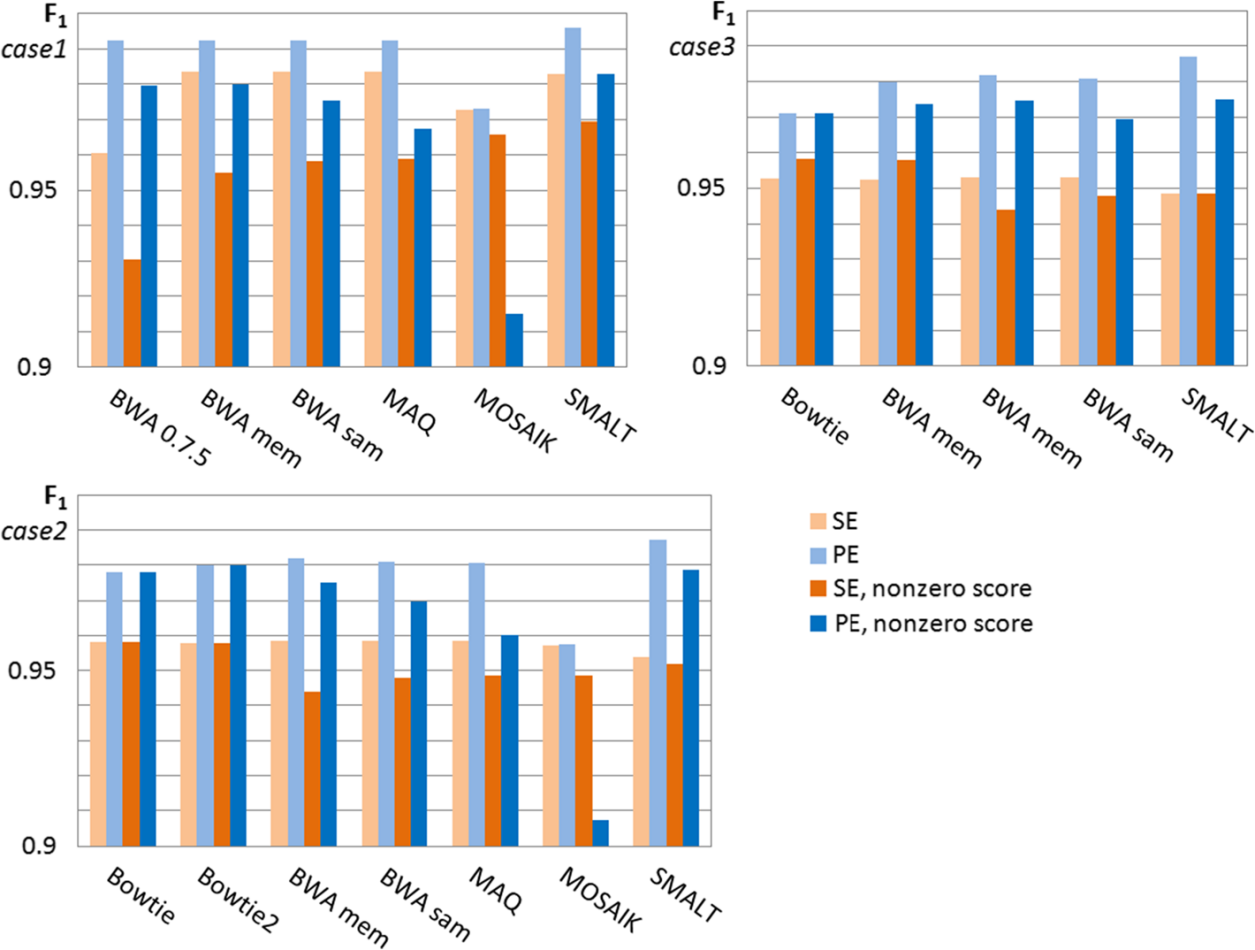
F1 scores. All reads – light colour bars, non-sero reads (‘reliable’) – dark colour bars

F_1_ scores for all cases are represented on Fig. 7.

Statistical results for all aligners look fairly even with respect to F_1_ values, and remarkably similar, despite ‘false‘peaks and gaps. This confirms our statement that the evaluation of aligners based only on confusion matrix (binary statistics) is incomplete.

The low value of F_1_ in the case of **MOSAIK** PE reads**,** especially ‘reliable‘, is explained by a relatively high level of lost reads. Besides, almost all its mismatched reads have non-zero score. Actually, it is a specific shortcoming of PE aligning for this tool. **BWA 0.7.5** has the highest level of lost SE reads, so it’s F_1_ for SE reads is the least.

### Mismatches statistics

Since all reads in an input sequence are full-defined, we can extract information about mismatches and their distribution. Please note that all mismatches arrive not because of non-perfect sequencing, but because of non-perfect aligning. Therefore, it is another estimation of the confidence of the mapping. To carry out this work, we developed our own auxiliary program **vAlign** [21].

### Percentage of reads with mismatches

**Fig. 8:**
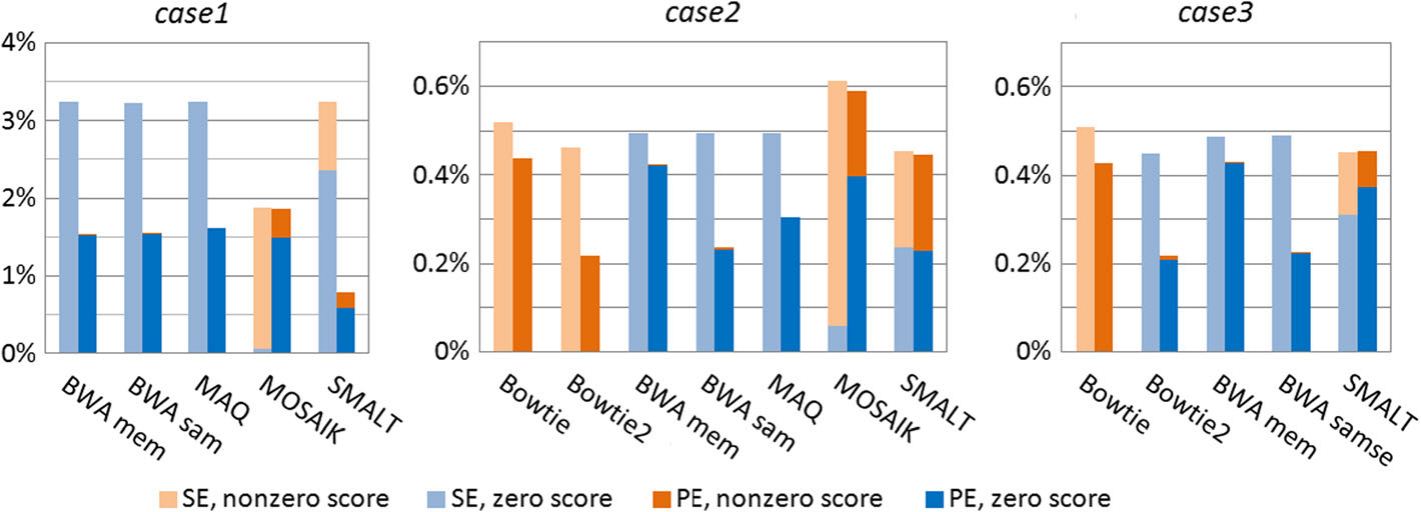
Percentage of non-zero-scored and zero-scored reads with detected mismatches. SE – pale histogram bars, PE – bright histogram bars

We counted reads within chromosome 1 with detected mismatches for each aligner. The percentage was calculated with respect to the total number of reads found on the chromosome 1 accordingly. For more information, zero-scored and non-zero-scored reads have been calculated separately. The results are represented in Fig. 8.

The first observation is a high percentage of mismatched reads in *case1* for all programs. In this case we can observe the most difference between SE and PE modes, except **MOSAIK’s** outcome.

### Specifics of distribution of mismatches along the reads

Among the studies devoted to aligners, we did not find information on the frequency distribution of the mismatches along the read. This issue has no practical significance for users, but may be of interest to aligner developers.

**Fig. 9:**
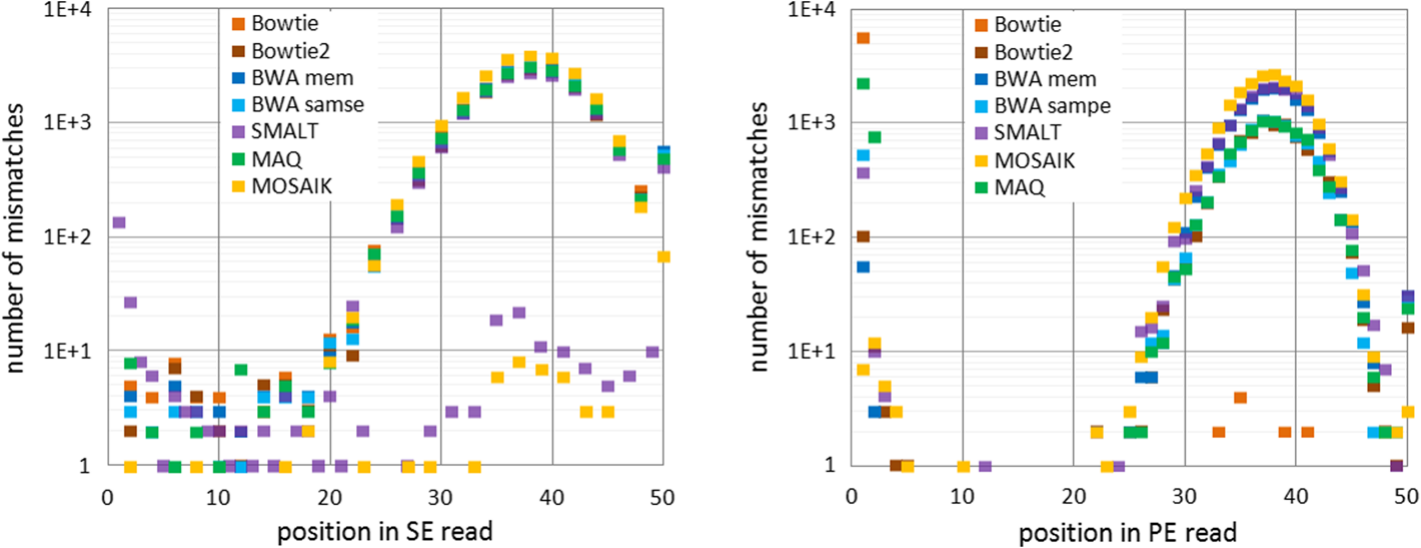
The frequency of mismatches depending on the position in the read, in log coordinate, *case2*

We summarized the mismatches in each nucleotide position for all the readers. The results for *case2* are shown in Fig. 9. Other cases look very similar.

Note that the odd positions in single-end reads did not contain mismatches for all programs, except **MOSAIK** and **SMALT** with very little count in odds.

The second remarkable feature is the observed increase in the number of mismatches in the second half of the reads, resembling a close to normal distribution. The maximum of the substitutions is at 38th position.

Also the large value is observed in the two first positions for most of aligners. This effect has a more localized appearance in PE mode.

The third interesting point is that these distributions generally are similar for all tools. On the whole, aligners utilize hashing algorithms or the Burrows-Wheeler transform to search exact matches. Nevertheless each tool uses its own optimization, which, as we see, leads to different results. However, this does not concern the distribution of mismatches and the appearance of gaps in *case2*.

### Summary of the results

Using an ‘ideal‘input sequence, we were expecting that aligning of reads from a single chromosome against a reference chromosome should be the simplest and, as a consequence, the best case for the aligners.

Indeed, 4 out of 6 tools showed F_1_ values exceeding 0.98 for SE reads, and 5 tools showed F_1_ values more than 0.99 for PE reads – these are very good statistical factors. Of these, **BWA mem, BWA samse** and **SMALT** showed very good coincidence with GSA in SE mode, while **MAQ** is perfect in both modes. Other programs generated a sequence that in some variants is distributed in a clearly different way from the sample. And this case really gives a gain in time of mapping from 20 to almost 50 times, as well as more than 10 times in following peak calling.

Mapping a single chromosome to a reference genome leads to system defects (gaps) in alignment, although 6 out of 7 tools showed a good and similar F_1_ values: about 0.96 for SE reads and 0.98 for PE reads.

When aligning a genome against a reference genome, all verified programs produced results statistically similar to the previous case. As for the distribution, **BWA samse** showed the best coincidence with GSA, in contrast to **BWA sampe**. **Bowtie** can be recognized as very good in both modes, despite the slightly lower value of F_1_. **Bowtie2** generated a little more of an uneven distribution. And both of **BWA mempe** and **SMALT** produced too many false enriched regions in PE mode.

## Discussion

### Distribution artefacts

As we mentioned before, all the sequences in the *case2* are very similar to each other. Consequently, features in the mapping algorithms that led to the obtained difference in cases *1* and *3*, did not appear in *case2*.

**Fig. 10:**
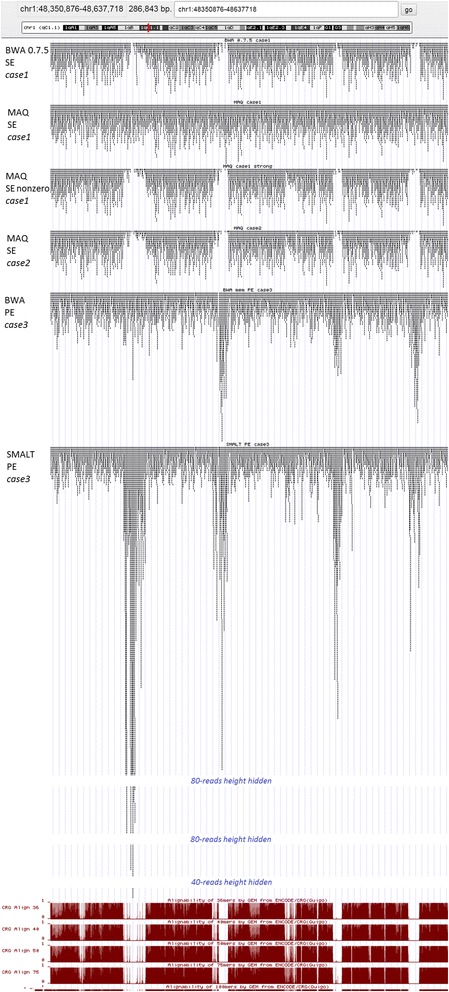
Fragment of alignments in comparison with low mappability tracks, UCSC genome browser

The difference in algorithms manifests itself in the way they process multi-reads, and therefore, first of all should be visible within the regions with low mappability [22]. Juxtaposition of the selected sequences with the genome mappability tracks confirmed this assumption, see Fig. 10.

First of all, **MAQ** demonstrates complete insensitivity to the low mappability regions in *case1*, and the synchronous coincidence of its ‘false gaps’ with such regions in *case2*. It is obvious that by referencing a single chromosome to the whole genome, the program first of all tries to best localize the next treated read throughout the whole reference, and matches not uniquely mapped on the first chromosome read to the different chromosomes, as well as other programs. As a result, only the unique hits remain on the first chromosome, which explains the high likeness of all alignments. This conclusion is also confirmed by a perfect correlation between **MAQ** *case2* and ‘reliable’ *case1*, with Pearson coefficient of 0.98.

Second, we can see that **BWA 0.7.5** almost replicates the low mappability regions by its ‘false gaps’ even in *case1*. At the beginning of the chromosome, the pattern is reversed: such regions correspond to its intensive ‘false peaks’, rapidly decreasing in amplitude with distance from the beginning. Obviously, the tool implements the relatively simple algorithm for mapping multi-reads, suggesting their location at the first suitable place, without analyzing the rest of sequence. Other aligners demonstrate more sophisticated approaches, with varying degrees of effectiveness.

A similar strategy is realized by **BWA** and **SMALT** in the PE mode in *case3*, but this time their ‘false peaks’ are distributed over the entire chromosome. At the same time these tools show a much better result for single end reads, especially **BWA samse** – actually it is a best SE aligner among the benchmarked in this practical case.

Thus, *case2* can be considered as a non-precise alternative to obtaining low mappability regions, as well as simply removing reads with the minimum admissible score.

### The artefacts in the distribution of mismatches

It possibly should be assumed that the observed effect is related to the seed-and-extend paradigm, followed by **Bowtie**, **BWA** and **MAQ**. In general all these software identify short matches as seeds, and then use the difference extending strategy. In this case, the default length of seed (28 bp for **Bowtie** and **MAQ**, and 32 bp for **BWA**) explains the small number of substitutions in the first half of the read. **SMALT** implements a different approach, but it also matches a read’s segment first. Unfortunately we did not find mention of the seeding in the **MOSAIK** descriptions. Such assumption is also not enough to explain the normal-shaped form of the distribution of mismatches in the second half of the read, as well as an increased level of substitution rate in the first two positions. This issue requires a deeper study of the aligning algorithms, which is beyond the scope of this article.

The question of the absence of mismatches on odd positions for SE reads also remains open.

## Conclusions

Our study showed that standard binary accuracy statistical measures are insufficient to assess the mapping performance. While having a comparable standard binary statistical metrics, all tested programs demonstrated significant differences in density distribution profiles. These differences are directly related to some shortcomings of the implemented matching algorithms, therefore, our modified CV can be considered as essential metric even in general-purpose alignments evaluation.

Note that gene regulatory regions in general have lower text complexity and corresponding lower mappability than protein-coding regions [23]. Biases in genomic nucleotide context and low complexity regions add noise to the mapping [24].

Observable deviations of the density of alignment from the sample have different significance. While ‘false’ peaks are completely eliminated by peak callers, the ‘false’ gaps can hide the real regions of interest. But ‘false peaks’ are formed due to ‘false gaps’, and vice versa.

Therefore, the best recommendation is to choose an aligner with the minimum value of both ‘head’ and ‘tail’ CVS. From this point of view and within the limits of the accepted conditions, in particular, while mapping a single chromosome, such tools are **MAQ**, **BWA mem** (in both modes) and **BWA samse** (for SE reads) work very good. When mapping a genome – **BWA samse** (for SE reads) and **Bowtie** (in both modes) are recommended.

We also showed some peculiarities of the distribution of mismatches along the reads. The pattern of error growth to the end of reads was commonly attributed to the accumulation of errors during sequencing. However, in our case there are no sequencing errors. This pattern seems to be common for all aligners, regardless of the various algorithms they are based on.
